# The utility of the Hopkins Verbal Learning Test (Chinese version) for screening dementia and mild cognitive impairment in a Chinese population

**DOI:** 10.1186/1471-2377-12-136

**Published:** 2012-11-07

**Authors:** Jing Shi, Jinzhou Tian, Mingqing Wei, Yingchun Miao, Yongyan Wang

**Affiliations:** 1 Department of Neurology, Dongzhimen Hospital, Beijing University of Chinese Medicine, Beijing, China; 2 Institute of Clinical Medicine, China Academy of Chinese Medical Sciences, Beijing, China

**Keywords:** Alzheimer’s dementia, Mild cognitive impairment, Hopkins verbal learning test, Screening test

## Abstract

**Background:**

The Hopkins Verbal Learning Test (HVLT) has been validated for detecting dementia in English-speaking populations. However, no studies have examined the Chinese version of the HVLT scale, and appropriate cut-off scores for dementia in the Chinese population remain unclear.

**Methods:**

631 subjects aged 60 and over were recruited at a memory clinic at Dongzhimen Hospital in Beijing. Of these, 249 were classified as exhibiting normal cognition (NC), 134 were diagnosed with mild cognitive impairment (MCI), 97 were diagnosed with Alzheimer’s disease (AD), 14 met the diagnosis for vascular dementia (VaD), and 50 were diagnosed with other types of dementia, including mixed dementia. The discriminative capacity of the HVLT total learning score, recognition score and total score were calculated to determine their sensitivity and specificity for detecting MCI, AD and other dementias, and various cut-off scores.

**Results:**

HVLT scores were affected by age, education and sex. The HVLT total learning score exhibited an optimal balance between sensitivity and specificity using a cut-off score of 15.5 for distinguishing AD and other types of dementia from NC using the ROC curve, with sensitivity of 94.7% for distinguishing AD and all types of dementia, and specificity of 92.5% for detecting AD and 93.4% for detecting all types of dementias. We stratified the AD and MCI groups by age, and calculated the validity in each age group. In the 50–64 years age group, when the cutoff score was 18.5, the sensitivity of 0.955 and specificity of 0.921 were obtained for discriminating the NC and AD groups, and in the 65–80 years group, and optimal sensitivity and specificity values (0.948 and 0.925, respectively) were obtained with a cutoff score of 14.5.

When the cutoff score was 21.5 in HVLT total recall, an optimal balance was obtained between sensitivity and specificity (69.1% and 70.7%, respectively) in distinguishing MCI from NC.

**Conclusion:**

A cut-off score of 15.5 in the HVLT total learning score led to high discriminative capacity between the dementia and NC groups. This suggests that the HVLT total learning score can provide a useful tool for discriminating dementia, but not MCI, from NC in clinical and epidemiological practice.

## Background

The increasing prevalence of dementia [1] is a major health problem among older adults [2]. A recent study reported that 5-9% of the Chinese population aged 65 years and over suffer from Alzheimer’s disease (AD) and 1-3% suffer from vascular dementia (VaD), with a total of 6 million patients with dementia in China at present [3]. With a rapidly ageing population in China, this prevalence rate is predicted to rise by more than 300% between 2001 and 2040 [4]. However, awareness, diagnosis and treatment of dementia remain relatively low in China. More than 48.8% of Chinese people regard dementia as a normal part of the ageing process, only 23.3% of patients with dementia seek medical advice, and 6.8% of patients are treated, compared with 50% in Europe. One study reported that the rate of misdiagnosis of dementia was 73.1%, and rates of treatment-seeking were 14.4%, 25.3% and 33.6% for mild, moderate, and severe dementia, respectively, between 1998 and 1999 [5]. Although the National Institute of Neurologic, Communicative Disorders and Stroke–Alzheimer Disease and Related Disorders Association (NINCDS-ADRDA) criteria have high sensitivity (up to 100%, with an average of 81% across studies), but relatively low specificity (an average of 70% across studies) for probable AD, based on class I–II studies with post-mortem confirmation [6]. Diagnostic accuracy is much lower in China, and most dementia is diagnosed in neurology clinics, where formal neuropsychological tests are lacking, because the diagnosis of dementia is expensive and procedures are time-consuming. At present, the diagnosis of dementia generally requires a medical history and neurological examination, laboratory blood studies, mental status assessment and formal cognitive tests, and computed tomography (CT) scans or magnetic resonance imaging (MRI) of the brain [6]. As such, there is a need to develop a useful test that can circumvent aspects of this process and guide physicians to accurate diagnosis. The Hopkins verbal learning test (HVLT) has been developed in an attempt to fulfill to this need [7]. Previous studies have shown that HVLT total learning score exhibits sensitivity and specificity of 87% and 98%, respectively, for discriminating patients with dementia from healthy controls [8], with an optimal discriminative capacity between mild cognitive impairment (MCI) and subjects with normal cognition (NC) [9]. However, all of these previous studies were performed in English-speaking countries, and no studies have examined the HVLT in the Chinese population. As such, the current study sought to establish the optimal cut-off score for discriminating NC, MCI and AD subjects in a Chinese population, and to evaluate the sensitivity and specificity for discriminating these three groups. In addition, we sought to determine whether HVLT scores are affected by social and demographic factors.

## Methods

### Subjects

A total of 631 subjects aged between 50 and 85 years were recruited for this study between January 2011 and September 2011 at a memory clinic at Dongzhimen Hospital in Beijing, China.

All participants underwent a routine clinical assessment, including detailed history, mental state examination, neurological examination, laboratory results (i.e. thyroid function, folic acid levels, vitamin B12, and routine blood tests, among others)and neuroimaging. Subjects were also assessed for the presence of other psychiatric disease that could influence cognition, including depressive disorder. Every participant underwent a complete neuropsychological assessment that mainly included the Instrumental Activities of Daily Living (IADL) [10] scale, the Hachinski Ischemia scale (HIS) [11], the Hamilton Depression Scale (HAMD) [12], the Adult Memory and Information Processing Battery (AMIPB) story recall [13], and the Clinical Dementia Rating (CDR) [14] score.

**Figure 1:**
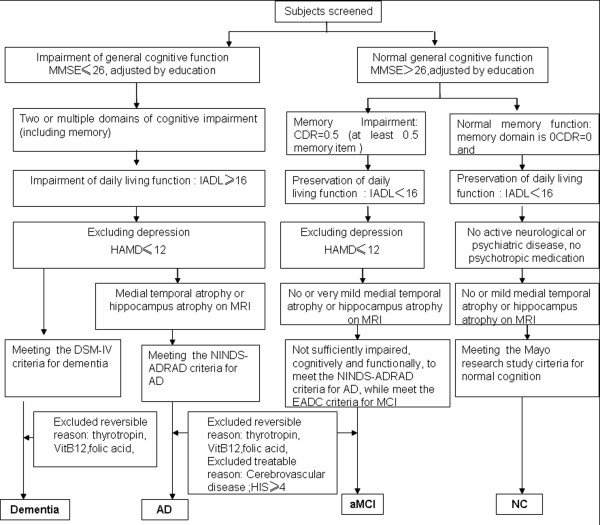
**Diagnostic algorithm for Alzheimer’s disease, amnestic MCI, and normal cognition.** Note: AD = Alzheimer’s disease; aMCI = amnestic mild cognitive impairment; CDR = clinical dementia rating scale; MMSE = Mini-mental State Examination; NC = normal cognition; IADL = Instrumental Activities of Daily Living; Hachinski Ischemia scale HAMD=Hamilton Depression Scale; EADC = Working Group of the European Consortium on Alzheimer's Disease.

The allocation of patients to different groups was mainly based on results of the mental state examination, neuropsychological assessment, laboratory results and neuroimaging. The diagnosis algorithm is shown in Figure 1.

Normal control subjects were identified according to the Mayo research study criteria [15]: (1) no active neurological or psychiatric disease, (2) no psychotropic medication, (3) the subjects may have medical disorders but neither they nor their treatment compromises cognitive function, the Mini Mental Status Examination (MMSE) [16,17] score >26 point (middle school), MMSE >22(primary school), MMSE >19 (Illiteracy), and a Clinical Dementia Rating (CDR) score of 0 [14].

The following criteria were used to define aMCI [18]: (1) memory complaints usually corroborated by an informant; (2) objective memory impairment (for age), Clinical Dementia Rating (CDR) score of 0.5, memory item score of 0.5; (3) normal general cognitive function, as determined by a clinician's judgment based on a structured interview with patients (a Mini-mental State Examination [MMSE] score of 24 to 30 for education) (cut-off scores: >19 for illiteracy, >22 for primary school, >26 for middle school and above) [16,17]; (4) no or minimal impairment in activities of daily living, as determined by a clinical interview with the patient and informant (an Instrumental Activities of Daily Living [IADL] score of <16) [10]; and (5) not sufficiently impaired, cognitively and functionally, to meet the NINCDS-ADRDA criteria for AD [19], as judged by an experienced AD research clinician. In addition, patients exhibited a score of ≤12 of the Hamilton Depression Scale (HAMD for 17 items) [12], ≤4 on the Hachinski Ischemia scale (HIS) [11], and no or minimal medial temporal atrophy (MTA) or hippocampal volume atrophy on the MRI scan [20]. All subjects had sufficient visual and auditory ability to complete the neuropsychological tests.

MCI exclusion criteria: (1) meeting criteria of dementia; (2) any major psychiatric disorder (*e.g*., DSM-IV-defined psychosis, major depression, bipolar disorder, or alcohol or substance abuse); (3) other neurological diseases including Parkinson's disease, or other neuropathy as verified by a formal clinical examination.

The diagnosis of dementia was based on the Diagnostic and Statistical Manual of Mental disorders, fourth edition [21],and the diagnosis of AD was based on the National Institute of Neurological Communicative Disease and Stroke (NINCDS) and Alzheimer's Disease and Related Disorders Association (ADRDA) criteria [19] for probable AD. Diagnosis of AD was based on clinical and neuropsychological assessments (Mini-mental State Examination: MMSE cutoff scores: ≤19 for illiteracy, ≤22 for primary school, ≤26 for middle school and above.); (2) two or multiple domains cognitive impairment; (3) Continued aggravation of memory and other cognitive functions; (4) absence of conscious disturbance; (5) impaired abilities of daily living, (IADL score ≥16); (6) without cerebrovascular disease, score of ≤4 on HIS; (7) and medial temporal atrophy (MTA) or hippocampus volume atrophy on the MRI scan; (8) Exclusion of other disease that may cause cognitive impairment.

AD exclusion criteria: (1) onset of unexpected apoplexy; (2) focal nervous system signs in the early stages of disease, for example, incomplete paralysis, anesthesia, dysfunctional visual field, and dystaxia ;(3) epileptic attack or gait disturbance in the early stages of disease; (4) any major psychiatric disorder (*e.g*., DSM-IV-defined psychosis, major depression, bipolar disorder, or alcohol or substance abuse); HAMD>12 (17 items).

For each subject, the HVLT was conducted by five examiners according to authors’ instructions [7]. The test included 12 words and the subject was asked to read aloud and freely recall immediately. This procedure was repeated three times, and total learning scores were calculated with the three free recall trails (range: 0–36). In addition, the three free recall trials were followed by a recognition trial consisting of 24 words, including 12 target list words; six ‘same’ categories of related non-target words; and six ‘other’ categories of unrelated words. Subjects then performed yes/no recognition. Recognition was scored by subtracting the number of false positive responses from the number of true positive responses during the recognition trial. HVLT total score was calculated by adding total learning and recognition scores.

Five examiners were asked to administer the HVLT, blinded to the subjects’ diagnosis. The HVLT test score had no impact on diagnosis decisions for either the patients with AD, dementia, aMCI or control subjects.

The study was undertaken in accordance with the principles of the Declaration of Helsinki. The protocol was approved by the Dongzhimen hospital institutional Ethics Committee. The patients and their caregivers provided written informed consent.

### Analysis

Statistical analyses were conducted using SPSS18.0 for Windows. The four groups were compared using non-parametric measures, and age, years of education, gender-ratio and race were compared between groups using Chi-squared tests.

The HVLT provides two basic summary scores. The HVLT total learning score is defined as the total score from the three free recall parts of the HVLT (=Trial1 + Trial2 + Trial3). The HVLT recognition score is calculated by subtracting the number of false positive responses from the number of true positive responses in the recognition trial. The HVLT total score was calculated by the sum of the total learning and the recognition scores,and was used to examine the discriminative ability of the test.

The ROC curves were calculated by plotting the sensitivity against the 1-specificity for each score on the HVLT total recall, recognition score and total score in discriminating between cases of dementia versus NC, between cases of AD versus NC and between cases of aMCI versus NC subjects. A multiple linear regression analysis was used to determine the influence of age, sex and education on HVLT score.

In addition, the positive predictive value (PPV) and negative predictive value (NPV) were calculated based on the prevalence in this sample.

## Results

A total of 631 subjects were enrolled. Three patients did not complete the neuropsychological test, 71 exhibited depression, 14 exhibited vascular cognitive impairment (VCI), 14 exhibited vascular dementia (VaD), 50 exhibited mixed dementia and other types of dementia, while the remaining 249 were classified as NC, 134 were diagnosed with aMCI, and 97 were diagnosed with AD. The ‘all types of dementia’ group (n = 161) included the AD only group (n = 97), the VaD only group (n = 14) and the mixed dementia group (n = 50). The diagnostic algorithm for AD and aMCI and NC is shown in Figure 1.

The demographic and neuropsychological characteristics of the different diagnostic categories are shown in Table 1. There were significant differences between the four groups. Subjects in the all types of dementia group and AD group were significantly older than those in the aMCI group (P = 0.000 and P = 0.000 for all types of dementia and AD groups, respectively), and had fewer years of education (P = 0.000 and P = 0.000 for the all types of dementia and AD groups, respectively). In addition, the aMCI group was significantly older than the NC group (P = 0.000) and had fewer years of education (P = 0.000).

**Table 1:** Demographic data and scores on neuropsychological tests

	**Diagnostic category**			
	**NC n=249**	**aMCI n=134**	**AD n=97**	**All type of dementia n=161**
Age	67.08(8.33)	69.89(8.23) **	71.08(8.43) **	71.0893(8.43) **
Education	12.89(3.30)	11.32(3.84) **	10.62(4.49) **	10.55(4.39) **
Sex(female/male)	158/91	76/58	54/43	81/80
Race(Han/others)	240/9	126/7	93/3	153/7
MMSE	28.41(1.50)	26.98(2.04) **	15.69(5.91) **△△	16.28(6.06)**△△
HVLT				
Trail 1	5.91(1.90)	4.48(1.69) **	1.46(1.81) **△△	1.57(1.76) **△△
Trail 2	8.54(2.05)	6.48(2.15) **	2.28(2.27) **△△	2.41(2.22) **△△
Trail 3	9.63(1.91)	7.21(2.65) **	2.55(2.59) **△△	2.71(2.45) **△△
Total learning	23.76(5.66)	18.04(5.93) **	6.09(6.23) **△△	6.37(5.91) **△△
Recognition	11.29(1.23)	10.32(2.18) **	3.42(5.38) **△△	3.95(5.52) **△△
HVLT total score	35.34(5.59)	28.49(6.67) **	9.65(10.54) **△△	10.54(10.19)**△△
HVLT total score/MMSE ratio	1.23(0.23)	1.05(0.26)	0.46(0.60)	0.50(0.56)
HAMD	3.78(3.30)	3.84(3.12) **	2.42(2.12) **△△	2.78(2.47) **△△

Notes: Data are presented as mean (standard deviation); NC = normal cognition; aMCI = amnestic mild cognitive impairment; AD = Alzheimer’s disease; MMSE = Mini-mental state examination; HVLT = Hopkins verbal learning test; HAMD = Hamilton Depression scale;**p < 0.001 compared to NC; p < 0.001 compared to aMCI.

The results revealed a significant difference between the four groups with respect to MMSE scores, HVLT total learning, HVLT recognition and HVLT total score (P = 0.000, P = 0.000). The all types of dementia group and AD group exhibited lower performance than the aMCI group in total learning (P = 0.000, P = 0.000, respectively), recognition (P = 0.000, P = 0.000, respectively), total score (P = 0.000, P = 0.000, respectively) and MMSE score (P = 0.000, P = 0.000, respectively) (see also Table 1), and aMCI patients exhibited poorer performance than the NC group regarding to all of the above tests. The ratio of HVLT total score / MMSE score exhibited a significant difference between the NC, MCI, AD and all types of dementia groups (P = 0.000). However, we found no significant differences between the AD group and the all types of dementia group in the HVLT total/MMSE ratio (P = 0.56). (P = 0.56).

There were no significant differences between the all types of dementia group and the AD group regarding age, sex, education, race, MMSE, and the HVLT total learning score, total scores (P > 0.05).

The ROC curves were produced by plotting the sensitivity against the 1-specificity for each score on the HVLT total learning, recognition score and total score in discriminating between cases of dementia versus NC, between AD cases versus NC and between cases of aMCI versus the NC group.

### Impact of demographic factors on HVLT

A multiple linear regression analysis was conducted with the HVLT total learning score as the dependent variable and age and years of education as independent variables. The results revealed significant effects (F = 54.607, P = 0.000), accounting for 38.7% of HVLT total learning scores, age (standardized coefficients = −0.253, P = 0.000) and years of education (standardized coefficients = 0.273, P = 0.000) impacted on HVLT total learning score.

### NC versus aMCI

**Figure 2:**
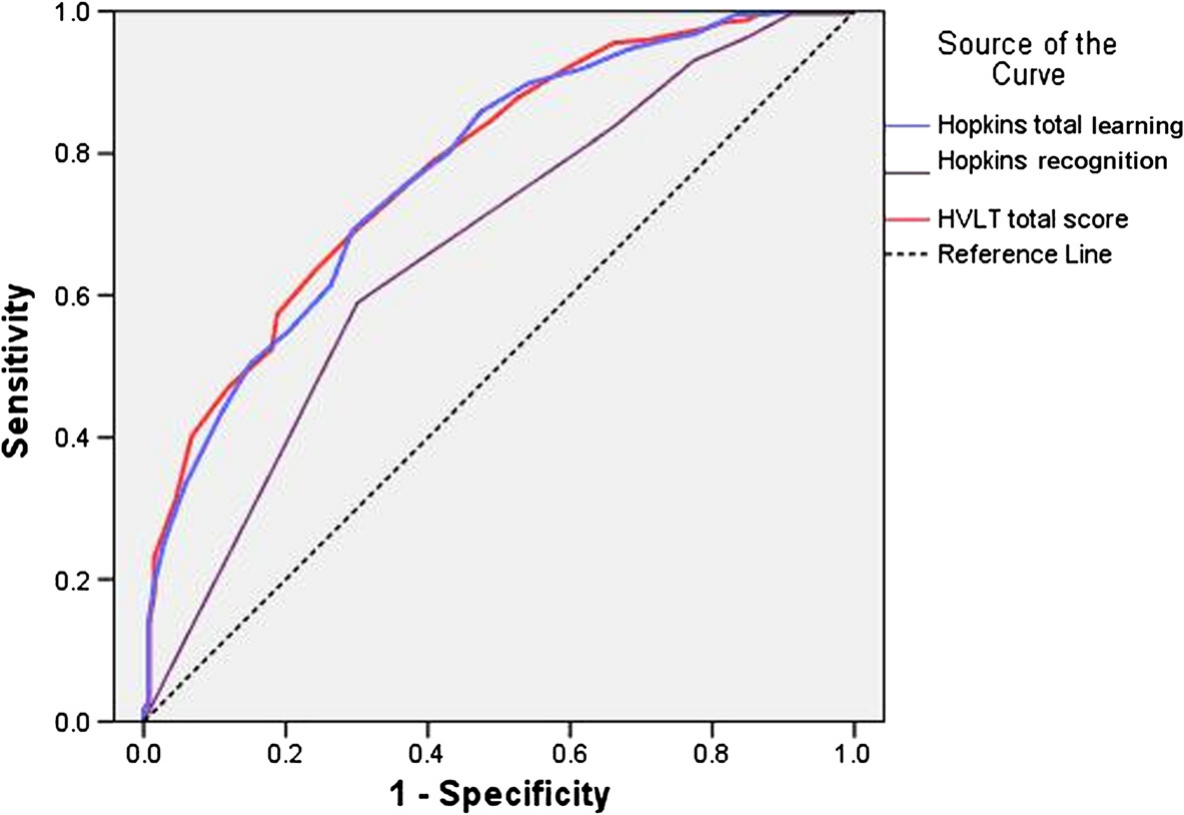
**ROC curve of the HVLT discriminate amnestic mild cognitive impairment from normal cognition.** Notes: HVLT = Hopkins verbal learning test, ROC = Receiver operating characteristic.

Figure 2 shows the sensitivity and specificity for distinguishing the aMCI group from the NC group, for total learning, recognition score and total HVLT, with different cut-off scores. The area under the curve (AUC) was 0.774 for the HVLT total learning score, and 0.79 for the HVLT total score. When the cutoff score was 21.5 in the HVLT total learning, an optimal balance was obtained between the sensitivity and specificity (69.1% and 70.7%, respectively) in distinguishing aMCI and NC. With a cut-off value of 32.5, the HVLT total score exhibited sensitivity and specificity of 68.7% and 70.7%, respectively.

The AUC was 0.666 for the HVLT recognition score, 0.589 for sensitivity, and 0.699 for specificity, when the cutoff score was 11.5. The sensitivity and specificity for HVLT recognition score were relatively low, indicating that this was not an ideal tool for discriminating aMCI from NC in our sample.

Because the HVLT total learning score was associated with age, the cut-off score was calculated in different age groups. In 50 to 64-year-old subjects, when the cut-off score was 23.5, optimal sensitivity (0.700) and specificity (0.718) values were achieved in discriminating the NC and aMCI groups. In addition, optimal sensitivity (0.776) and specificity (0.562) values were obtained when the cutoff score was 18.5 in the 65–80 year-old patients.

### NC versus AD and all types of dementia

Optimum sensitivity and specificity of HVLT total learning score, HVLT recognition score and HVLT total score for discriminating AD and all types of dementia from NC were determined by the ROC curve analysis using the most appropriate cut-off scores. The total AUC for total learning score was 0.976 for discriminating AD, and 0.982 for detecting all types of dementia. The total AUC for the HVLT total score was 0.982 for discriminating AD, and 0.986 for detecting all types of dementia. The total learning score exhibited optimal sensitivity and specificity using a cut-off score of 15.5 for detecting NC and AD and all types of dementia, with similar sensitivity for detecting AD and all types of dementia (94.7% for both), and specificity of 92.5% for AD, and 93.4% for all types of dementia. The HVLT total score exhibited better overall discrimination of AD and all types of dementia patients, using a cut-off score of 25.5, with 93.5% specificity and 95.5% sensitivity for detecting AD, and with 93.9% specificity and 96.0% sensitivity when the optimal cutoff score was 26.5 for detecting all types of dementia.

In the 50–64 years age-group optimal values for sensitivity (0.955) and specificity (0.921) were obtained for discriminating the NC and AD groups when the cutoff score was 18.5. Optimal values for sensitivity (0.948) and specificity (0.925) were obtained with a cutoff score of 14.5 in the 65–80 years age-group for discriminating the NC and AD groups.

The HVLT recognition scores for discriminating all types of dementia and AD were also calculated. The AUC was 0.96 for detecting AD, and 0.935 for detecting all types of dementia.

The sensitivity, specificity, PPV and NPV of HVLT total learning cut-off scores for different prevalence rates of all types of dementia are shown in Table 2. In our sample, the prevalence of all types of dementia was 39.19%, and when the cut-off score the HVLT total learning score was 15.5, the PPV was 0.90, and NPV was 0.95. The PPV was impacted by the baseline prevalence, with PPV increasing when prevalence increased.

**Table 2:** Sensitivity and specificity of HVLT total learning scores to discriminate all types of dementia and probabilities of dementia (PPV) and probabilities of no dementia (NPV) at different baseline rates

**All type of dementia**			**PPV/NPV at different base rate**			
**HVLT Cut-off**	**Sensitivity**	**Specificity**	**5%**	**10%**	**15%**	**20%**
<10.50	1.000	0.762	0.18/1.00	0.32/1.00	0.43/1.00	0.51/1.00
<11.50	0.996	0.781	0.19/0.99	0.34/0.99	0.45/0.99	0.53/0.99
<12.50	0.996	0.841	0.25/099	0.41/0.99	0.53/0.99	0.61/0.99
<13.50	0.967	0.854	0.26/0.96	0.42/0.96	0.54/0.96	0.62/0.96
<14.50	0.963	0.901	0.34/0.96	0.52/0.96	0.63/0.96	0.71/0.96
<15.50*	0.947	0.934	0.43/0.95	0.61/0.95	0.72/0.95	0.78/0.95
<16.50	0.919	0.947	0.48/0.92	0.66/0.92	0.75/0.92	0.81/0.92
<17.50	0.898	0.947	0.47/0.90	0.65/0.90	0.75/0.90	0.81/0.90
<18.50	0.858	0.974	0.63/0.87	0.79/0.87	0.85/0.87	0.89/0.87
<19.50	0.801	0.974	0.62/0.83	0.77/0.83	0.84/0.83	0.89/0.83
<20.50	0.756	0.980	0.67/0.80	0.81/0.80	0.87/0.80	0.90/0.80
<21.50	0.691	0.980	0.65/0.76	0.79/0.76	0.86/0.76	0.90/0.76

* indicate the cutoff with optical sensitivity and specificity.

## Discussion

In the current study, we calculated the optimal cut-off scores in our sample for detecting aMCI, AD, and all types of dementia from NC controls. The results revealed that the HVLT alone works equally well for distinguishing both AD and all types of dementia. We investigated the optimal discriminative capacity of the HVLT for distinguishing between NC controls and cases with AD and all types of dementia. The results revealed that the optimal balance between sensitivity and specificity for detecting the NC from AD and all types of dementia with the HVLT total learning score was obtained with a cutoff score of 15.5. Other studies have reported similar results, achieving much higher sensitivity and specificity (0.96 and 0.80, respectively) with a cut-off score of 18–19 resulting in sensitivity and specificity of 0.96 and 0.80, respectively [22]. The difference in cut-off scores may be because of a difference between the sample in the present experiment and this previous study. In present study, we enrolled dementia subjects with moderate to severe cognitive impairment, and the mean MMSE score was 16.28. In contrast, a previous study included mild dementia subjects and mild cognitive impairment, reporting a mean MMSE score of 21.8 [22].

As a screening test for dementia, sensitivity is likely to be more important than specificity for detecting a greater number of patients in the general population. However, among dementia patients, specificity may be more important than sensitivity when examining subjects with severe dementia. In the current sample, sensitivity of 85.8% and specificity of 91.0% were obtained when the cut-off score was 18.5 for discriminating mild AD (CDR = 1). When the cut-off score was 15.5 for discriminating moderate AD (CDR = 2), sensitivity of 94.7% and specificity of 100% were obtained. These results suggest that the HVLT has higher specificity for discriminating AD from NC.

However, the HVLT revealed a relatively low discrimination capacity for detecting aMCI from NC. In the current study, when the optimal cut-off score was 21.5 for detecting aMCI from NC, we obtained low sensitivity (69.1%) and specificity (70.7%) respectively. These values are lower than those in another study showing that the HVLT has specificity of 95% and sensitivity of 79% in detecting MCI when the optimal cutoff was 25.5 [9]. This discrepancy may be related to the small sample size of the previous study, which only recruited 21 aMCI patients and 98 normal controls, substantially smaller than the sample in the present study. Another possible explanation for the difference between the current findings and previous reports is that global cognition, measured by MMSE, was substantially lower in the current study compared to previous reports in the aMCI group. The lower HVLT total learning scores in aMCI patients in the current study may have led to lower optimal cutoff scores.

In our study sample, multiple linear regression analysis revealed that demographic variables (i.e., sex, age and education) affected HVLT total recall. Other studies have reported a positive relationship between HVLT scores and years of education [23]. Higher levels of education were associated with a higher HVLT total learning score. Cognitive reserve may have also affected the current results. Since patients with a higher educational level exhibit greater cognitive reserves, more pathology may be required before memory begins to be affected [24]. In addition, age had an inverse impact on the HVLT total learning score. When controlling for education and sex, older subjects exhibited lower HVLT total scores. On the contrary, a number of studies have reported no relationship between HVLT total learning scores and age [8,9,23]. The HVLT total learning score was also influenced by sex, with females exhibiting lower sores than males when controlling for age and education. This may because of the different physiological characteristics of gender, or as a result of environmental and social factors [25].

The MMSE is widely used to assess global cognition in clinical settings for many years, and the MMSE is recommended as a screening tool for early dementia [6]. A previous study reported that when the cut-off score for the MMSE was 24.5, the sensitivity and specificity were 0.89 and 0.91,respectively, for detecting dementia in subjects with higher educated [26]. However, a number of studies have reported that the MMSE has lower discriminating capacity than HVLT for detecting dementia [8,9,23]. In addition, a ceiling effect has been reported for the MMSE, with 30% of controls achieving perfect scores [8]. The MMSE score is also affected by age and education [26]. Taken together, these findings indicate that the HVLT may be more suitable for screening purposes.

The PPV and NPV of HVLT total learning score in the current study were relatively high. This finding suggests that subjects with negative results on the HVLT total learning score may not require further neuropsychological evaluations.

## Conclusion

The current results revealed that the HVLT total learning score and total score can discriminate well between cases of dementia and NC controls, suggesting that it is a useful tool in clinical and epidemiological practice. When the cut-off score of the HVLT total learning score was 15.5, it was capable of a high level of discrimination between dementia and normal controls. When the HVLT was used to discriminate aMCI patients from NC controls, discrimination capacity was relatively low. When an optimal cut-off score of 21.5 was used for detecting aMCI from NC, sensitivity of 69.1% and specificity of 70.7% were obtained. Hence, further study may be needed to detect the use of HVLT for aMCI.

## Abbreviations

AD: Alzheimer’s disease; MCI: Mild cognitive impairment; CDR: Clinical dementia rating scale; MCI: Mild cognitive impairment; MMSE: Mini-mental State Examination; NC: Normal cognition; PPV: Positive predictive value; NPV: Negative predictive value; ROC: Receiver operating characteristic analysis curve; HVLT: Hopkins verbal learning test.

## Misc

Jing Shi and Jinzhou Tian contributed equally to this study.

## Competing interests

There are no competing interests.

## Authors’ contributions

JT and JS designed the study, planned analysis of the data, and wrote and reviewed the manuscript. MW, YM, JT and JS carried out the neuropsychological assessment, and JT and JS were principal investigators for this study and finalized the manuscript. YYW was reviewed the design of the trial protocol. All authors read and approved the final manuscript.

## Pre-publication history

The pre-publication history for this paper can be accessed here:

http://www.biomedcentral.com/1471-2377/12/136/prepub
